# Association between abnormal glycoprotein and diabetic peripheral neuropathy in patients with type 2 diabetes mellitus

**DOI:** 10.3389/fendo.2025.1697737

**Published:** 2025-12-05

**Authors:** Dongmei Zhu, Deyue Kong, Qian Li, Hemin Jiang, Ziyang Shen

**Affiliations:** 1 Department of Pediatric Surgery, Nantong First People’s Hospital, Nantong, China; 2 Department of Endocrinology, Nanjing First Hospital, Nanjing Medical University, Nanjing, China; 3 Department of Endocrinology and Metabolism, The First Affiliated Hospital with Nanjing Medical University, Nanjing, China

**Keywords:** abnormal glycoprotein, diabetic peripheral neuropathy, nerve conduction study, restricted cubic spline analysis, receiver operating characteristic

## Abstract

**Background and objectives:**

To investigate the association between serum abnormal glycoprotein (AP) and diabetic peripheral neuropathy (DPN) in patients with type 2 diabetes mellitus (T2DM).

**Materials and methods:**

This cross-sectional study enrolled 476 patients with T2DM. DPN was diagnosed using the Toronto Clinical Neuropathy Score (TCNS) and nerve conduction studies. The association between AP and DPN was evaluated using multivariable logistic regression, restricted cubic spline analysis (RCS), and receiver operating characteristic (ROC) curve analysis.

**Results:**

Serum AP levels were significantly higher in patients with DPN than in those without (P < 0.001). After adjusting for multiple confounders, elevated AP emerged as an independent risk indicator for DPN (OR = 1.024, 95% CI: 1.012-1.036). A non-linear relationship was observed, with a marked increase in DPN risk when AP levels exceeded an inflection point of 119.628 μm². Combining AP with clinical variables significantly enhanced predictive accuracy for DPN, increasing the area under the curve (AUC) from 0.686 to 0.805.

**Conclusions:**

Elevated serum AP represents a novel and independent risk indicator for DPN in patients with T2DM. Its integration into clinical practice may facilitate early detection for DPN.

## Introduction

Type 2 diabetes mellitus (T2DM) is a chronic metabolic disorder characterized by persistent hyperglycemia. Its incidence in children and adults is rising rapidly worldwide. Diabetic peripheral neuropathy (DPN), one of the most prevalent chronic complications of diabetes mellitus, affects approximately 50% of patients over the course of their disease (1). It constitutes a major cause of disability, leading to neuropathic pain, foot ulcerations, and non-traumatic lower limb amputations, thereby severely diminishing patients’ quality of life and imposing a substantial economic burden on healthcare systems (2, 3). Despite its clinical significance, the early and accurate diagnosis of DPN remains challenging. Current diagnostic methods primarily rely on clinical symptom assessment and nerve conduction studies (NCS), the latter of which is invasive and expensive and exhibits limited sensitivity for detecting early-stage nerve fiber damage (1, 4). Consequently, there exists an urgent unmet clinical need for novel, sensitive, and non-invasive biomarkers for the early detection of DPN.

The pathophysiology of DPN is complex, with chronic hyperglycemia serving as the primary driver. Sustained hyperglycemia instigates a cascade of metabolic derangements, in which abnormal protein glycosylation represents a key mechanism underlying diabetic complications (5). This non-enzymatic reaction between excess glucose and proteins leads to the formation of advanced glycation end products, which accumulate in nerve tissues and contribute to neuronal damage by inducing oxidative stress, inflammation, and microvascular dysfunction (6, 7). Altered levels and structures of various glycoproteins have been consistently observed in diabetes and are closely associated with its complications, suggesting that a marker reflecting systemic protein glycosylation status may hold substantial diagnostic value (1, 5).

Abnormal glycoprotein (AP) is a complex glycoprotein initially identified as a serum biomarker for various malignancies (8–10). Its diagnostic utility in oncology derives from the characteristic changes in glycosylation patterns and expression levels that occur during carcinogenesis, reflecting altered cellular metabolism (7, 11). Importantly, these alterations are not exclusive to cancer. Pathological states that disrupt normal cellular metabolism, such as the chronic hyperglycemia and inflammatory microenvironment characteristic of diabetes, can theoretically induce similar modifications in protein glycosylation. This hypothesis is supported by a recent study reporting significantly elevated AP levels in patients with type 2 diabetes, even in the absence of concurrent malignancy, thereby linking AP directly to the diabetic metabolic state (12). This finding provides a compelling rationale for exploring AP beyond its traditional role as a tumor marker.

Given that abnormal glycosylation constitutes a fundamental pathological link between diabetes and DPN and that AP serves as a sensitive indicator of systemic glycosylation changes, we hypothesized that AP levels may be elevated in patients with DPN. To date, no studies have investigated the relationship between serum AP levels and the presence of DPN. Therefore, this study aimed to assess the expression levels of serum AP in patients with DPN and to evaluate its potential as a novel, non-invasive biomarker for diagnosing this debilitating complication.

## Research design and methods

### Study participants

This cross-sectional study was conducted at the Department of Endocrinology, Nanjing First Hospital, from November 2024 to May 2025. Based on the American Diabetes Association diagnostic criteria (2024) for T2DM, we consecutively recruited patients with T2DM from the inpatient departments. Inclusion criteria comprised a confirmed diagnosis of T2DM and available AP laboratory results. Exclusion criteria included (1) type 1 diabetes or other specific types of diabetes; (2) severe hepatic dysfunction (ALT or AST ≥ 3-fold the upper limit of normal) or renal dysfunction (eGFR < 45 mL/min/1.73 m^2^); (3) active malignant tumors; (4) history of other diseases known to cause peripheral neuropathy, such as chronic alcoholism, vitamin B12 deficiency, or exposure to neurotoxic agents; (5) age > 85 years, presence of mental illness, or inability to complete required examinations; and (6) pregnancy or lactation. A total of 476 patients with T2DM were enrolled in the final analysis. The study protocol was approved by the Institutional Review Board of Nanjing First Hospital (KY20250811-KS-03). The study was conducted in accordance with the principles of the Declaration of Helsinki. All participants provided written informed consent prior to any study-related procedures.

### DPN assessment

DPN was assessed using the Toronto Clinical Neuropathy Score (TCNS) and nerve conduction studies. The TCNS is a validated and reliable scale for diagnosing of DPN (13). Nerve conduction studies were performed on all participants by an experienced technician blinded to the participants’ clinical status. The evaluation includes the conduction velocity, amplitude, and latency of motor nerves and sensory nerves. Participants with a TCNS score ≤ 5 and normal nerve conduction findings were classified as being without DPN. Participants with DPN were defined by the combination of a TCNS score > 5 combined with abnormal findings on nerve conduction studies.

### Clinical and laboratory assessments

Baseline demographic data, including age, sex, height, weight, duration of diabetes, smoking status, alcohol consumption, history of hypertension, coronary heart disease (CHD), and use of antidiabetic agents, were collected through patient interviews and a review of medical records. Smoking status is defined as “never,” “ever” (quit smoking >1 year ago), or “current” (currently smoking). Alcohol consumption status is defined as “never,” “ever” (quit drinking >1 year ago), or “current” (any regular drinking within the past 12 months). Body mass index (BMI) was calculated as weight (kg) divided by height squared (m²). Resting systolic and diastolic blood pressure (SBP/DBP) were measured by trained nurses.

Following an overnight fast of at least 8 h, venous blood samples were collected for biochemical analysis. Glycated hemoglobin A1c (HbA1c) was determined using high-performance liquid chromatography (D-10; Bio-Rad, Hercules, CA, USA). Fasting plasma glucose (FPG), creatinine (Cr), uric acid (UA), total cholesterol (TC), triglycerides (TG), high-density lipoprotein cholesterol (HDL), and low-density lipoprotein cholesterol (LDL) were measured using automatic colorimetric assays (Hitachi 7180, Tokyo, Japan). The estimated glomerular filtration rate (eGFR) was calculated using the Chronic Kidney Disease Epidemiology Collaboration (CKD-EPI) equation. Serum concentrations of AP were quantified using a commercially available detection kit (Zhejiang Ruisheng Medical Technology, Ltd., Cixi, China) according to the manufacturer’s instructions.

### Statistical analysis

All statistical analyses were performed using SPSS software (version 23, IBM Corp., Armonk, NY, USA) and R software (version 4.5.1, R Foundation for Statistical Computing, Vienna, Austria). Continuous variables were presented as mean ± standard deviation (SD) for normally distributed data or median (interquartile range), for non-normally distributed data, as assessed by the Shapiro–Wilk test. Categorical variables were expressed as frequencies and percentages (n, %). Differences between the DPN and non-DPN groups were assessed using the Welch’s t-test or Mann–Whitney U test for continuous variables and the chi-square test or Fisher’s exact test for categorical variables, as appropriate. Welch’s ANOVA or Kruskal–Wallis H test were used to explore the difference among AP quartile groups. Spearman’s correlation analysis was conducted to evaluate the association between serum AP levels and other clinical and laboratory parameters. Multivariable logistic regression models were employed to examine the independent association of AP with DPN. To model and visualize the potential non-linear relationship between serum AP concentrations and the risk of DPN, restricted cubic spline (RCS) analysis with five knots was performed within the logistic regression framework. Receiver operating characteristic (ROC) curve analysis was used to evaluate the diagnostic performance of AP alone and in combination with other clinical variables. A two-sided P-value < 0.05 was considered statistically significant.

## Results

### Characteristics of the study participants

Of the 476 enrolled participants, 85 (17.9%) were diagnosed with DPN. The clinical and laboratory characteristics are presented in **Table 1**. Compared with those without DPN, patients with DPN were significantly older, had a longer duration of diabetes, exhibited poorer glycemic control, and displayed lower DBP. Serum AP levels were significantly higher in patients with DPN than in those without DPN. Additionally, patients with DPN had significantly lower serum ALT, AST, eGFR, TG, and LDL levels, along with higher serum Cr levels. The prevalence of CHD and hypertension was significantly higher in the DPN group. As anticipated, motor nerve conduction velocities (MNCVs) of the median and common peroneal nerves, as well as the sensory nerve conduction velocity (SNCV) of the median nerve, were significantly slower in patients with DPN. Furthermore, patients with DPN demonstrated higher rates of alpha-glucosidase inhibitor use and insulin therapy.

**Table 1:** Baseline characteristics of patients with versus without diabetic peripheral neuropathy.

Variables	Patients without DPN (n=391)	Patients with DPN (n=85)	P‐value
Male n,(%)	219 (56.0)	49 (57.6)	0.789
Age (years)	58.17 ± 12.27	69.88 ± 8.68	<0.001
Diabetes duration (years)	8 (2,15)	10 (8,20)	<0.001
BMI (kg/m2)	25.28 ± 3.64	24.97 ± 3.54	0.462
Hypertension n,(%)	191 (48.8)	55 (64.7)	0.008
CHD n,(%)	45 (11.5)	25 (29.4)	<0.001
SBP (mmHg)	133.29 ± 19.23	136.64 ± 22.9	0.213
DBP(mmHg)	84.09 ± 11.99	79.65 ± 11.75	0.002
HbA1c (%)	8.69 ± 1.56	9.11 ± 1.8	0.048
ALT (U/L)	19 (15,29)	16 (11.5,22.5)	<0.001
AST (U/L)	19 (16,24)	18 (14,22)	0.018
Cr (μmol/L)	67.28 ± 18.59	73.51 ± 20.18	0.01
FPG (mmol/L)	7.08 ± 2.66	7.8 ± 2.7	0.027
TC (mmol/L)	4.53 ± 1.25	4.24 ± 1.17	0.051
TG (mmol/L)	1.37 (0.94,1.94)	1.13 (0.88,1.57)	<0.001
HDL (mmol/L)	1.01 ± 0.24	1.04 ± 0.27	0.264
LDL (mmol/L)	2.6 ± 0.89	2.38 ± 0.85	0.036
eGFR (mL/min/1.73m2)	95.55 ± 17.82	82.38 ± 18.81	<0.001
AP (μm²)	84.94 ± 21.1	102.84 ± 27.39	<0.001
Smoking n,(%)			0.117
Never	295 (75.4)	63 (74.1)	
Current	91 (23.3)	18 (21.2)	
Ever	5 (1.3)	4 (4.7)	
Drinking n,(%)			0.046
Never	354 (90.5)	76 (89.4)	
Current	37 (9.5)	7 (8.2)	
Ever	0 (0)	2 (2.4)	
Antidiabetic agents, n (%)			
Metformin	187 (47.8)	41 (48.2)	0.945
Sulfonylureas	65 (16.6)	18 (21.2)	0.316
Alpha glucosidase inhibitors	67 (17.1)	24 (28.2)	0.018
SGLT2is	108 (27.6)	29 (34.1)	0.231
DPP4is	71 (18.2)	11 (12.9)	0.248
Thiazolidinediones	19 (4.9)	3 (3.5)	0.779
Insulin	103 (26.3)	37 (43.5)	0.002
GLP-1RA	48 (12.3)	7 (8.2)	0.291
Nerve conduction velocity			
L,median nerve MNCV (m/s)	62.34 ± 8.97	55.53 ± 10.27	<0.001
R,median nerve MNCV (m/s)	63.03 ± 13.53	55.71 ± 14.87	<0.001
L,peroneal never MNCV (m/s)	51.05 ± 10.87	45.65 ± 16.01	0.004
R,peroneal never MNCV (m/s)	49.79 (47.06,53.45)	40.47 (37.18,44.88)	<0.001
L,median nerve SNCV (m/s)	62.54 ± 13.55	54.04 ± 16.84	<0.001
R,median nerve SNCV (m/s)	59.44 ± 10.45	51.75 ± 19.16	<0.001

The data are presented as mean ± SD, numbers (%), or medians (interquartile ranges).

### Correlation of AP with clinical parameters

**Figure 1:**
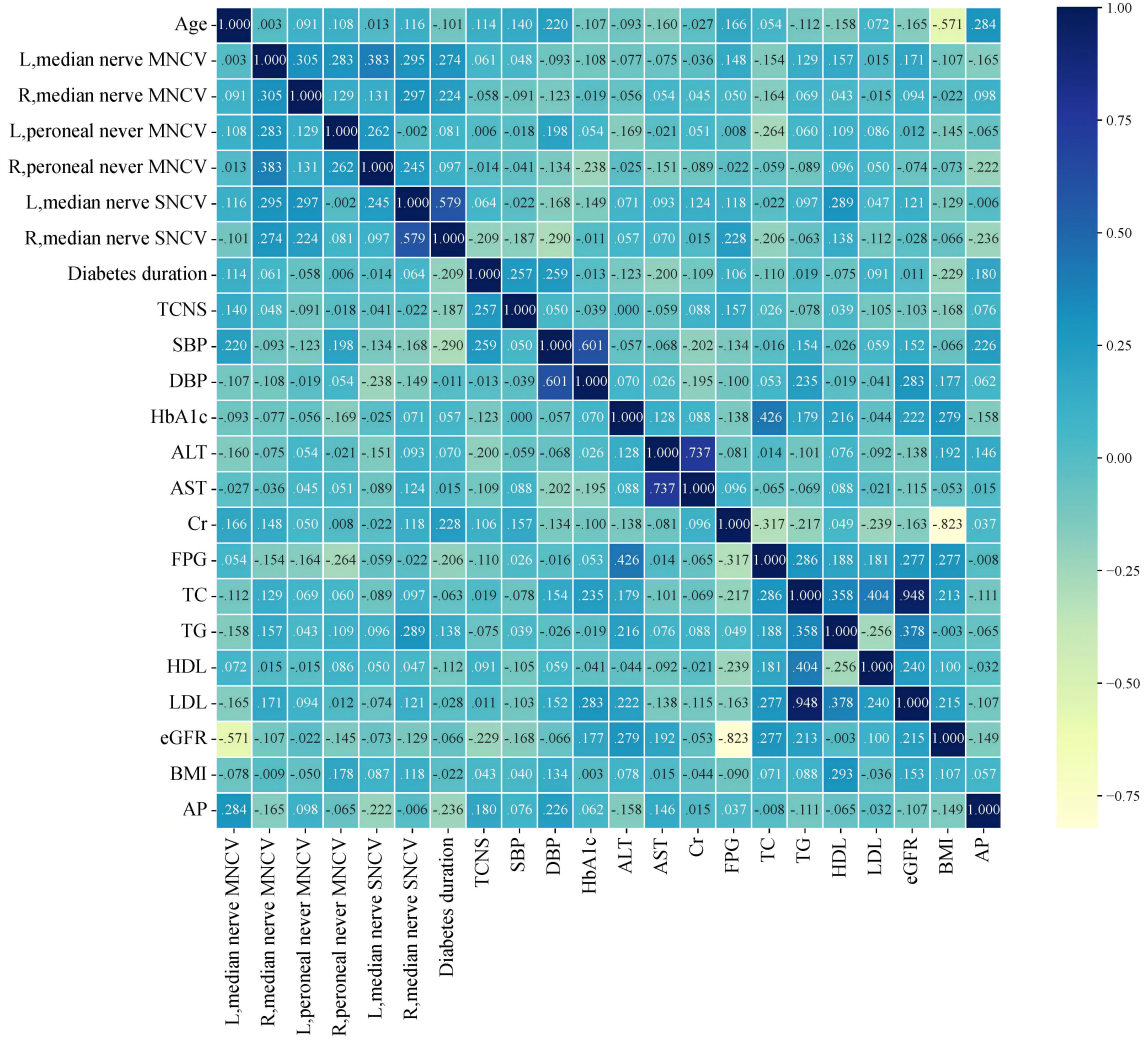
The heatmap depicts the relationship between the AP and the other variables in DPN patients.

As shown in **Figure 1**, Spearman’s correlation analysis in the patients with DPN revealed that AP was significantly and negatively correlated with nerve conduction velocities, including right peroneal nerve MNCV and right median nerve SNCV. Conversely, AP was positively correlated with age and SBP. Additionally, AP exhibited a weak correlation with HbA1c, although this did not reach statistical significance.

### Association between AP levels and DPN

The association between AP levels and DPN was first examined by treating AP as a continuous variable in multivariable logistic regression models (**Table 2**). This association remained significant in the fully adjusted model (model III), which controlled for age, diabetes duration, HbA1c, eGFR, BMI, and sex. In this final model, each 1-μm² increase in AP was linked to an odds ratio of 1.024 (95% CI 1.012–1.036).

**Table 2:** Multivariable logistic regression analysis exploring relationships between AP and diabetic peripheral neuropathy.

Variables	Model I	P-value	Model II	P-value	Model III	P-value
	OR (95% CI)		OR (95% CI)		OR (95% CI)	
AP	1.033 (1.023,1.045)	<0.001	1.024 (1.013,1.037)	<0.001	1.024 (1.012,1.036)	<0.001
Q1	Ref		Ref		Ref	
Q2	2.001 (0.833, 5.53)	0.13	1.989 (0.771,5.471)	0.164	1.933 (0.746,5.343)	0.185
Q3	4.269 (1.935,10.453)	<0.001	3.544 (1.5,9.216)	0.006	3.352 (1.411,8.747)	0.009
Q4	5.55 (2.557,13.454)	<0.001	3.13 (1.323,8.125)	0.013	2.827 (1.181,7.403)	0.025

Model I unadjusted. Model II adjusted for age, diabetes duration, HbA1c, and eGFR. Model III adjusted for age, diabetes duration, HbA1c, eGFR, BMI, and sex.

**Figure 2:**
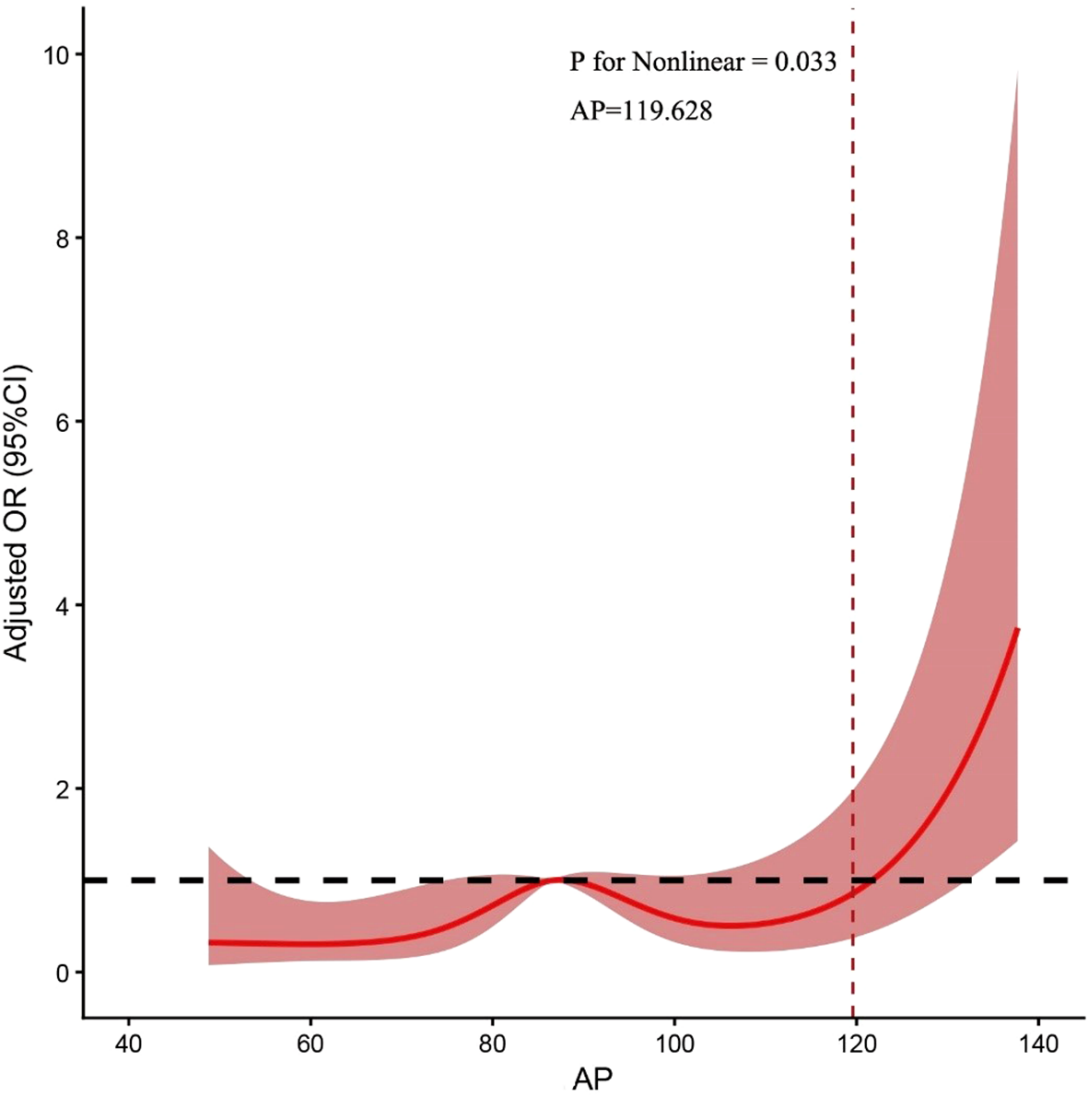
Restricted cubic spline of the linear trends between the AP and DPN, adjusted for age, diabetes duration, HbA1c, eGFR, BMI, and sex.

To further investigate the correlation between serum AP levels and DPN, RCS regression was applied in a multivariate adjusted model. This analysis revealed a significant non-linear association between AP levels and the risk of DPN (**Figure 2**). A threshold effect analysis was subsequently performed using a two-piecewise logistic regression model. An inflection point in the association of AP with DPN was identified at a level of 119.628 μm². Below this threshold, there was no statistically significant association between AP and the risk of DPN. However, for AP levels above this inflection point, each 1-μm² increase was associated with a 12.5% increase in the risk of DPN (OR 1.125, 95% CI 1.054–1.2, P<0.001).

When analyzing AP levels by quartiles, participants in the highest quartile (Q4) consistently exhibited a significantly greater risk of developing DPN compared with the reference group (Q1). As shown in **Table 3**, participants with elevated AP levels exhibited a higher prevalence of DPN, TCNS, age, and diabetes duration compared with those in the lower quartiles. Additionally, left median nerve MNCV and both median nerve SNCV were lower in participants with higher AP levels. Finally, lower eGFR, LDL, and DBP were observed in Q4.

**Table 3:** Baseline characteristics of the study population according to AP quartile group. The data are presented as mean ± SD, numbers (%), or medians (interquartile ranges).

Variables	AP quartiles				P‐value
	Q1 (42.63-70.44 μm²)	Q2 (70.44-86.61 μm²)	Q3 (86.61-105.04 μm²)	Q4 (105.04-178.15 μm²)	
Sample size (n)	119	119	119	119	
DPN n, (%)	8 (6.7)	15 (12.6)	28 (28.6)	34 (28.6)	<0.001
Male n,(%)	64 (53.8)	67 (56.3)	69 (58)	68 (57.1)	0.939
Age (years)	55.17 ± 12.83	57.94 ± 12.89	61.8 ± 10.69	66.14 ± 10.88	<0.001
Diabetes duration (years)	8.48 ± 7.53	9.54 ± 8.22	10.56 ± 8.1	11.81 ± 10.83	0.033
BMI (kg/m2)	25.53 ± 3.27	24.98 ± 3.76	25.11 ± 3.94	25.29 ± 3.48	0.641
Hypertension n,(%)	53 (44.5)	60 (50.4)	58 (48.7)	75 (63)	0.029
CHD n,(%)	14 (11.8)	12 (10.1)	26 (21.8)	18 (15.1)	0.053
SBP (mmHg)	136.39 ± 24.29	130.1 ± 16.59	134.24 ± 20.11	134.83 ± 17.62	0.061
DBP(mmHg)	86.78 ± 13.39	81.98 ± 10.12	83.33 ± 12.45	81.1 ± 11.38	0.003
HbA1c (%)	9 ± 1.63	8.7 ± 1.57	8.74 ± 1.55	8.62 ± 1.68	0.315
ALT (U/L)	20 (14,29)	18 (14,26)	19 (14,30)	20 (14,27)	0.897
AST (U/L)	19 (16,24)	18 (16,23)	19 (15,24)	19 (16,23)	0.68
Cr (μmol/L)	67.6 ± 17.84	66.34 ± 19.09	69.1 ± 19.13	70.53 ± 19.92	0.374
FPG (mmol/L)	7.48 ± 2.97	7.11 ± 2.59	7.23 ± 2.64	7 ± 2.51	0.582
TC (mmol/L)	4.6 ± 1.38	4.31 ± 1.22	4.61 ± 1.23	4.39 ± 1.11	0.152
TG (mmol/L)	1.27 (0.98,1.89)	1.37 (0.93,1.97)	1.38 (0.96,2.1)	1.25 (0.82,1.58)	0.231
HDL (mmol/L)	1.01 ± 0.25	0.98 ± 0.23	1 ± 0.25	1.06 ± 0.24	0.115
LDL (mmol/L)	2.64 ± 0.97	2.44 ± 0.83	2.71 ± 0.88	2.46 ± 0.83	0.035
eGFR (mL/min/1.73m2)	97 ± 18.98	96.73 ± 17.97	91.49 ± 18.62	87.58 ± 17.68	<0.001
AP (μm²)	60.32 ± 6.64	77.94 ± 4.33	94.84 ± 4.91	119.43 ± 13.76	<0.001
Smoking n,(%)					0.104
Never	84 (70.6)	91 (76.5)	88 (73.9)	95 (79.8)	
Current	34 (28.6)	28 (23.5)	27 (22.7)	20 (16.8)	
Ever	1 (0.8)	0 (0.0)	4 (3.4)	4 (3.4)	
Drinking n,(%)					0.47
Never	110 (92.4)	103 (86.6)	110 (92.4)	107 (89.9)	
Current	9 (7.6)	15 (12.6)	8 (6.7)	12 (10.1)	
Ever	0 (0)	1 (0.8)	1 (0.8)	0 (0)	
Antidiabetic agents n, (%)					
Metformin	58 (48.7)	59 (49.64)	63 (52.9)	48 (40.3)	0.256
Sulfonylureas	18 (15.1)	17 (14.3)	20 (16.8)	28 (23.5)	0.236
Alpha glucosidase inhibitors	17 (14.3)	20 (16.8)	24 (20.2)	30 (25.2)	0.163
SGLT2is	31 (26.1)	38 (31.9)	34 (28.6)	34 (28.6)	0.81
DPP4is	22 (18.5)	17 (14.3)	27 (22.7)	16 (13.4)	0.219
Thiazolidinediones	9 (7.6)	5 (4.2)	6 (5)	2 (5.5)	0.215
Insulin	33 (27.7)	34 (28.6)	33 (27.7)	40 (33.6)	0.731
GLP-1RA	17 (14.3)	18 (15.1)	10 (8.4)	10 (8.4)	0.207
TCNS	1 (0,3)	2 (0,4)	2 (0,5)	2 (1,6)	0.001
Nerve conduction velocity					
L,median nerve MNCV (m/s)	63.21 ± 12.36	61.49 ± 8.68	60.62 ± 8.32	59.18 ± 7.91	0.018
R,median nerve MNCV (m/s)	61.67 ± 12.55	63.01 ± 15.48	61.02 ± 11.87	61.21 ± 15.93	0.721
L,peroneal never MNCV (m/s)	51.62 ± 14.88	49.62 ± 8.14	50.28 ± 13.48	48.82 ± 10.79	0.4
R,peroneal never MNCV (m/s)	49.92 ± 5.51	53.47 ± 23.29	49.09 ± 6.96	49.59 ± 21.29	0.242
L,median nerve SNCV (m/s)	61.5 ± 11.85	64.68 ± 18.56	59.65 ± 11.42	58.24 ± 14.58	0.017
R,median nerve SNCV (m/s)	59.37 ± 10.23	60.8 ± 12	56.75 ± 13.65	55.36 ± 14.29	0.006

### Subgroup analysis

**Figure 3:**
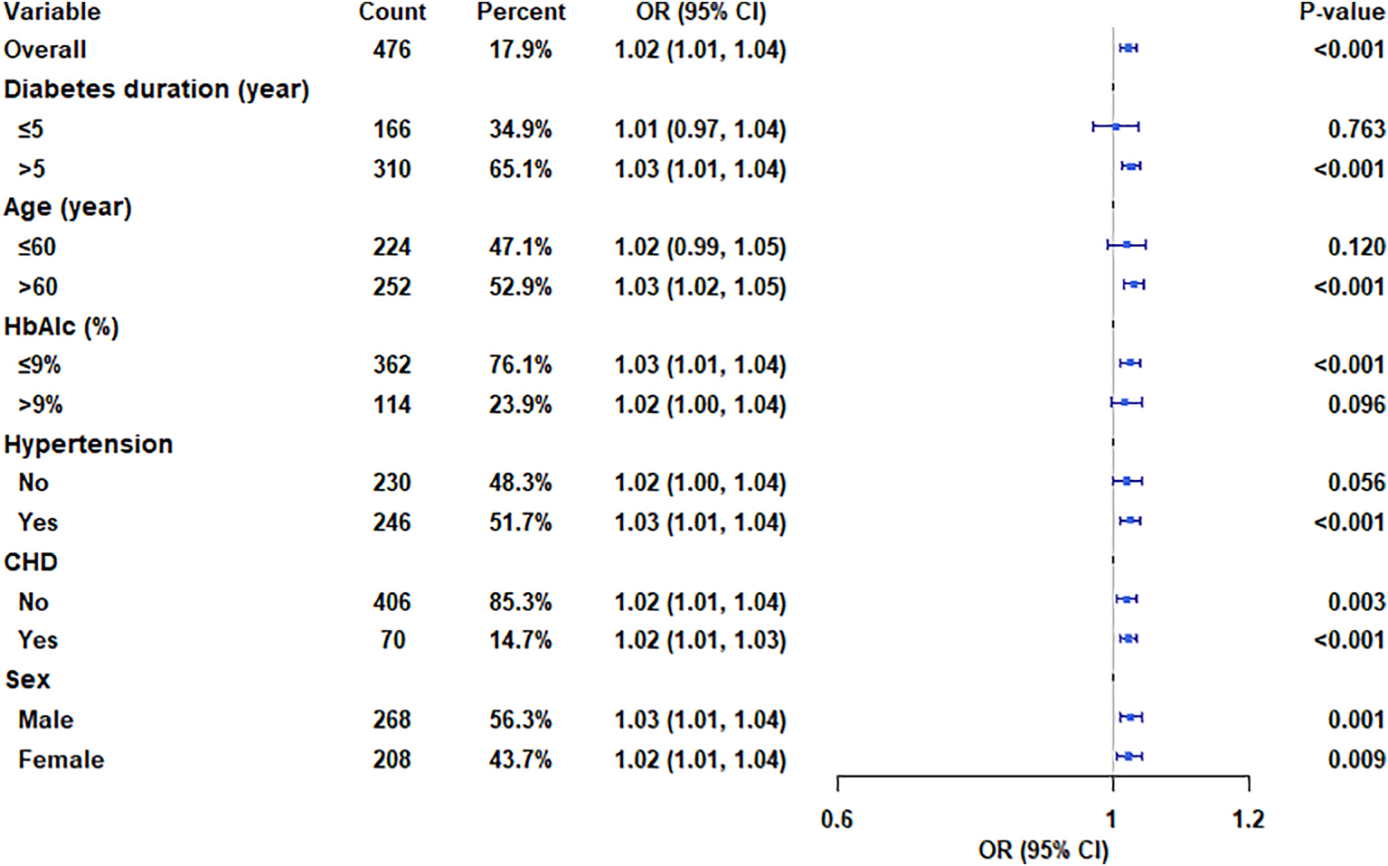
Relationship between AP and DPN in subgroup populations stratified by age, sex, HbA1c, diabetes duration, and the presence of hypertension and CHD, adjusted for relevant confounders.

We further explored the association between AP and DPN in several clinically relevant subgroups (**Figure 3**). The models were adjusted for age, diabetes duration, HbA1c, eGFR, BMI, and sex. The results indicate that the positive association between continuous AP levels and DPN risk was consistent across most subgroups, including those defined by sex, age (>60 years), diabetes duration (>5 years), HbA1c levels (≤ 9%), and the presence of hypertension or CHD. No significant interactions were observed, suggesting the association is robust.

### Performance of AP alone and combined indicators in predicting DPN

**Figure 4:**
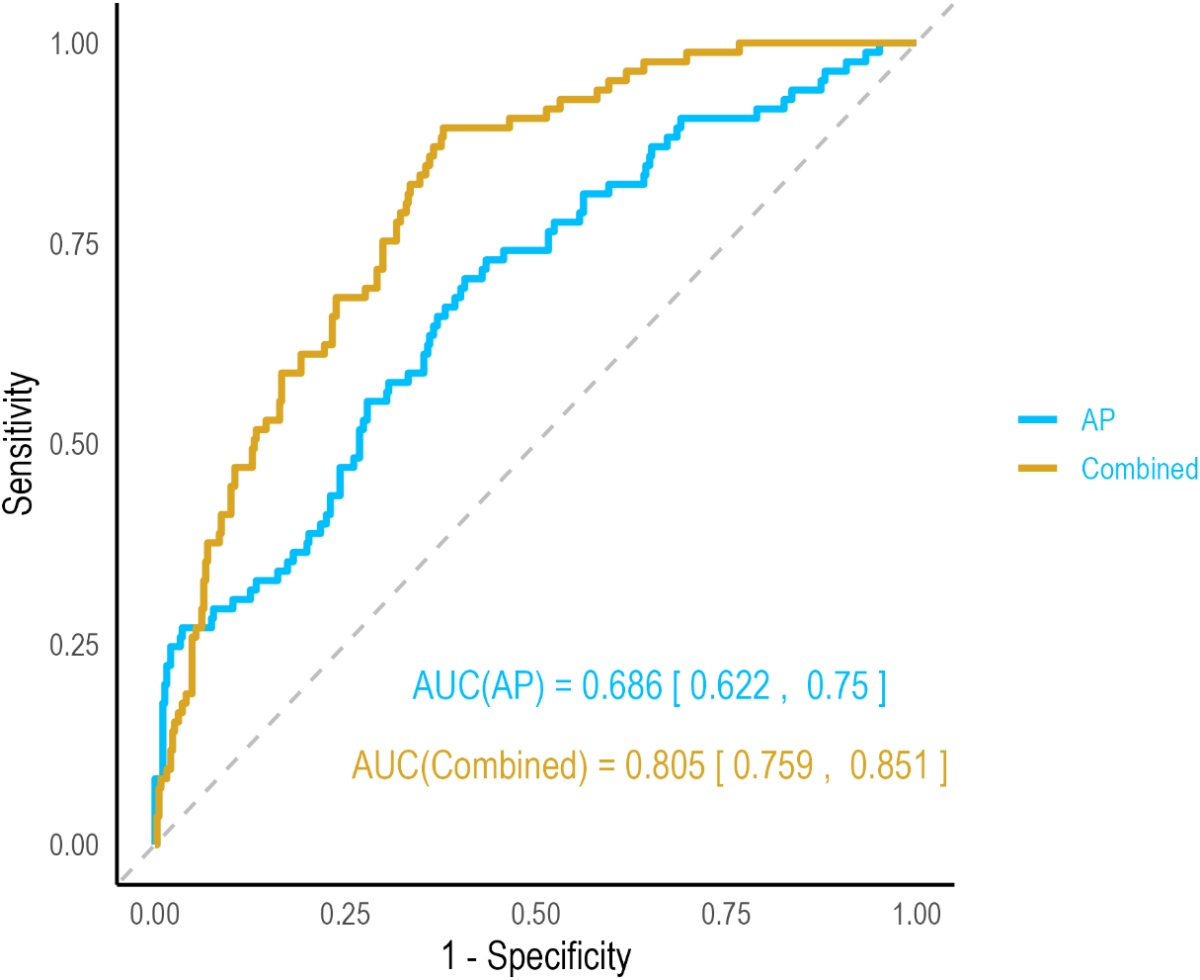
ROC curves for DPN comparing AP alone and the combined model.

ROC curve analysis was employed to assess the predictive capacity of AP alone and a combined model for DPN (**Figure 4**). The area under the curve (AUC) for AP alone in predicting DPN was 0.686, with an optimal cutoff of 90.23μm², yielding a sensitivity of 70.6% and a specificity of 59.3%. To enhance predictive accuracy, a combined model was developed incorporating AP with simple clinical variables: age, diabetes duration, and sex. The AUC for this combined model improved significantly to 0.805. These findings illustrate the substantial improvement in predictive performance when AP is integrated with clinical risk factors.

## Discussion

This study provides the first evidence of a significant association between serum AP levels and DPN in patients with T2DM. While AP was initially recognized in oncology as a biomarker reflecting abnormal protein glycosylation, its diagnostic potential in DPN, a non-neoplastic disease primarily associated with metabolic dysregulation, has remained unexplored (14, 15). Our findings indicate that serum AP levels are significantly elevated in patients with DPN compared with those without, and this elevation is inversely correlated with nerve conduction velocities. After adjusting for multiple confounding factors, elevated AP levels remained a stable and independent risk indicator for DPN.

A core finding of this study is the non-linear relationship between AP and DPN risk, an important yet often overlooked pattern in biomarker research (16, 17). Through RCS analysis, we identified a critical risk inflection point at 119.628 μm². Below this threshold, the association between AP and DPN risk was not statistically significant; however, once this point was exceeded, the risk of DPN increased sharply with rising AP levels. This threshold effect suggests the existence of a “metabolic tipping point” in the pathophysiology of DPN (18).

In terms of diagnostic performance, AP demonstrated moderate efficacy (AUC = 0.686), comparable with other known DPN biomarkers such as neurofilament light chain (19). Notably, when AP was combined with standard clinical risk indicators, the model’s diagnostic power improved substantially, with the AUC increasing to 0.805. This result indicates that AP provides unique pathological information about cumulative metabolic damage that is independent of traditional risk factors, thereby significantly enhancing the accuracy of DPN diagnosis.

This association is not a mere conceptual transfer but is grounded in the established biological consensus that aberrant glycosylation is a shared pathological feature of numerous chronic diseases, including both cancer and diabetes (7, 20).

In the pathophysiology of diabetes, the core mechanism underlying abnormal protein modification is the non-enzymatic glycation reaction resulting from chronic hyperglycemia, which culminates in the formation of advanced glycation end products by directly cross-linking with long-lived proteins in nerve tissue, such as myelin and cytoskeletal proteins, thereby disrupting their structure and function (21–23), and by binding to their specific receptor to activate downstream inflammatory and oxidative stress pathways (24). This mechanism links AP to two critical elements in the DPN pathological process: inflammation and structural damage.

Multiple studies have confirmed that systemic inflammatory markers, such as C-reactive protein, are associated with DPN risk (25). The serum neurofilament light chain, a recently prominent biomarker for axonal damage, directly reflects neuronal death or degeneration when elevated (26). Compared with these markers, AP may associate with this inflammation and structural damage, as it is hypothesized to be closely linked with hyperglycemia, the core pathology of diabetes.

This observation suggests a potential pathophysiological timeline for DPN biomarkers that could guide future research: First, glycation stress emerges as the earliest, specific pathological signal; subsequently, this stress may contribute to neuroinflammation and irreversible structural damage; finally, this structural damage may manifest as clinical functional deficits, such as abnormal postural sway and sudomotor dysfunction (27, 28).

However, this study has several limitations that must be acknowledged. First, the cross-sectional design establishes an association but cannot confirm causality or predict the future incidence of DPN. We cannot determine whether elevated AP levels was associated with DPN, nor if the pathological state of DPN was linked with AP accumulation. To address this, future prospective cohort studies are essential to measuring baseline AP levels in individuals without DPN and following them over time to establish the predictive value and temporal relationship of AP. Second, the study participants were recruited from a single medical center, which may limit the generalizability of the findings to other ethnic groups or populations with different clinical characteristics. Third, because current commercial kits cannot distinguish specific AP subtypes, future studies may need to use techniques such as mass spectrometry to identify which particular AP subtypes are most closely associated with neuropathy. Therefore, validation of these findings in larger, multicenter cohorts is necessary to ensure their broad applicability.

In conclusion, this study identifies AP as a potent and independent risk indicator for DPN. By demonstrating a non-linear relationship and a clear inflection point, these findings indicate that AP could help shift the management paradigm for DPN from reactive treatment of established disease to preemptive prevention in high-risk individuals, thereby potentially improving long-term patient outcomes.

## Data Availability

The original contributions presented in the study are included in the article/supplementary material. Further inquiries can be directed to the corresponding authors.
